# Identification and Investigation of *Drosophila* Postsynaptic Density Homologs

**DOI:** 10.4137/bbi.s2010

**Published:** 2008-11-03

**Authors:** Faith L.W. Liebl, David E. Featherstone

**Affiliations:** 1 Southern Illinois University Edwardsville, Department of Biological Sciences, Edwardsville, IL, U.S.A; 2 University of Illinois at Chicago, Department of Biological Sciences, Chicago, IL, U.S.A

## Abstract

AMPA receptors are responsible for fast excitatory transmission in the CNS and the trafficking of these receptors has been implicated in LTP and learning and memory. These receptors reside in the postsynaptic density, a network of proteins that links the receptors to downstream signaling components and to the neuronal cytoskeleton. To determine whether the fruit fly, *Drosophila melanogaster*, possesses a similar array of proteins as are found at the mammalian PSD, we identified *Drosophila* homologs of 95.8% of mammalian PSD proteins. We investigated, for the first time, the role of one of these PSD proteins, Pod1 in GluR cluster formation at the *Drosophila* neuromuscular junction and found that mutations in *pod1* resulted in a specific loss of A-type receptors at the synapse.

## Introduction

The majority of neurotransmission in the mammalian central nervous system uses glutamate as a neurotransmitter. One type of ionotropic glutamate receptor, AMPA receptors (AMPARs), is responsible for fast excitatory transmission in the CNS. The regulated delivery and insertion of AMPARs receptors has been implicated in long term potentiation (LTP, for review see Malinow and Malenka, 2002) and contextual fear learning (Hu et al. 2007; Matsuo et al. 2008). Therefore, the mechanisms that govern AMPAR expression and trafficking are of considerable interest.

AMPARs are tetramers composed of GluR1-4 (Hollmann and Heinemann, 1994; Monoghan and Wenthold, 1997; Gereau and Swanson, 2008). Although AMPARs may be synthesized in dendrites (Ju et al. 2004), most AMPAR mRNA is located in the neuronal cell body suggesting that AMPARs must be transported to their synaptic destinations (Esteban, 2003). There is some evidence that kinesins mediate the cellular trafficking of AMPAR-containing vesicles along the microtubule cytoskeleton. The heavy chain of kinesin directly interacts with GRIP (Setou et al. 2002), which binds to the AMPAR subunits GluR2 and GluR3 (Dong et al. 1997; Srivastava et al. 1998). GluR2 and GRIP also associate with liprin-α (Wyszynski et al. 2002), which interacts with KIF1 (Shin et al. 2003). Vesicles containing AMPARs must be transferred from microtubules to actin filaments before their final delivery into dendritic spines. This process may be mediated by the motor protein, myosin Vb (Lise et al. 2006). Trafficking of receptors to the synapse is mediated by a family of transmembrane regulator proteins (TARPs) (Tomita et al. 2003; Tomita et al. 2004; Tomita et al. 2005; Nicoll et al. 2006; Ziff, 2007) that may also influence AMPAR kinetics (Milstein et al. 2007).

AMPARs are dynamically regulated at the synapse. For example, transient stimulation of NMDA receptors sufficient to produce LTP results in the rapid insertion of AMPARs into the postsynaptic membrane (Liao et al. 1995; Liao et al. 1999; Liao et al. 2001;Poncer and Malinow, 2001) possibly from recycling endosomes (Park et al. 2004). This *de novo* insertion of receptors is dependent upon the interaction between the AMPAR subunit, GluR1 and the scaffolding protein, SAP97 (Hayashi et al. 2000). At synapses, AMPARs are part of dense protein networks called postsynaptic densities (PSD), which are located opposite from presynaptic release sites. The molecular composition of the PSD has been characterized using biochemical approaches, mass spectrometry, and proteomics (Kennedy, 1998; Husi and Grant, 2001; Jordan et al. 2004; Peng et al. 2004; Boeckers, 2006; Collins et al. 2006; Dosemeci et al. 2007) revealing a complex structure composed of hundreds of proteins. The complexity of the interactions between proteins suggests that perturbations of many PSD proteins could affect AMPAR trafficking or localization.

We sought to determine whether the fruit fly, *Drosophila melanogaster*, possesses a similar array of proteins as are found at the mammalian glutamatergic PSD. The *Drosophila* genome encodes 21 putative ionotropic glutamate receptor subunits, including homologs of mammalian NMDA, AMPA, kainate, and delta receptor subunits (Sprengel et al. 2001). The *Drosophila* neuromuscular junction (NMJ) is glutamatergic making it similar in composition and function to mammalian central synapses (Collins and DiAntonio, 2007). The receptors at the NMJ are classified non-NMDA receptors. Similar to their mammalian homologs, *Drosophila* GluRs are tetramers that contain three essential subunits including GluRIIC (Marrus and DiAntonio, 2004), GluRIID (Featherstone et al. 2005), and GluRIIE (Qin et al. 2005) along with either GluRIIA (Schuster et al. 1991) or GluRIIB (Petersen et al. 1997). These two receptor types, A-type (which contain GluRIIA, -IIC, -IID, and -IIE but not -IIB) or B-type (which contain GluRIIB, -IIC, -IID, and -IIE but not -IIA), are differentially expressed and clustered (Marrus and DiAntonio, 2004; Schmid et al. 2008) and interact with distinct components of postsynaptic density (Chen and Featherstone, 2005; Chen et al. 2005).

As in mammals, *Drosophila* glutamate receptors form postsynaptic tetramers that mediate fast synaptic transmission (DiAntonio, 2006), and NMDA receptors are required for learning (Xia et al. 2005, Lin, 2005; Wu et al. 2007). This suggests that glutamate receptor (GluR) function may be largely conserved, but it remains unknown whether mechanisms of glutamate receptor trafficking and anchoring are also conserved. The use of an evolutionarily simpler system could facilitate the understanding of molecular functions and relationships between proteins involved in GluR trafficking. We found that 95.8% of mammalian PSD proteins have *Drosophila* homologs. We investigated, for the first time, the role of one of these PSD proteins, Pod1, in GluR cluster formation at the NMJ and found that mutations in *pod1* resulted in a specific loss of A-type receptors at the synapse.

## Materials and Methods

### Bioinformatics

We searched the literature for proteins that regulate AMPAR, KARs, or reside in the PSD. Mammalian protein sequences were extracted from the National Center for Biotechnology Information (http://www.ncbi.nlm.nih.gov/). The mammalian sequences used were either mouse, rat, or human. The amino acid sequence obtained was compared with annotated proteins in *Drosophila* using FlyBase’s BLAST (http://flybase.bio.indiana.edu/blast/). Gene expression patterns were retrieved from the Berkeley Drosophila Genome Project Expression Pattern database (http://www.fruitfly.org/cgi-bin/ex/insitu.pl).

### Antibodies and immunocytochemistry

For immunocytochemistry and microscopy, animals were dissected and fixed for 30–60 min in either Bouin’s fixative (when GluR antibodies were used), or 4% paraformaldehyde in PBS (for Pod1 labeling). Third instar larvae were dissected and fillet preparations were pinned down in Sylgard lined Petri dishes. All dissections were done in *Drosophila* standard saline (135 mM NaCl, 5 mM KCl, 4 mM MgCl, 1.8 mM CaCl, 5 mM TES, 72 mM sucrose) at RT. Mouse monoclonal anti-GluRIIA (Iowa Developmental Studies Hybridoma Bank, Iowa City, IA) was used at 1:100. Rabbit polyclonal anti-GluRIIB and anti-GluRIIC were gifts from Aaron DiAntonio (Washington University, St. Louis, MO) and were used at 1:2000 and 1:5000, respectively. Guinea pig polyclonal anti-Pod1 was a gift from Yuh-Nung Jan (University of California, San Francisco) and was used at 1:1000. Fluorescently conjugated anti-HRP (Jackson Immunoresearch Labs, West Grove, PA) was used at 1:100. Goat anti-rabbit, goat anti-mouse, or goat anti-guinea pig fluorescent (FITC or TRITC) secondary antibodies (Jackson Immunoresearch Labs, West Grove, PA) were used at 1:400. The 6/7 NMJ of abdominal hemisegments A3 or A4 were used for all studies. Confocal images were obtained using an Olympus FV500 laser-scanning confocal microscope. Image analysis and quantification was performed using ImageJ and Adobe Photoshop software.

### Electrophysiology

All electrophysiology was performed on the ventral body wall muscle 6. Larval recordings were performed on third instar larvae 110–120 hr AEL. Muscle 6 was voltage-clamped at −60 mV. Standard two-electrode voltage clamp techniques were used, as previously described (Liebl et al. 2005). Data were acquired and analyzed using a Gene clamp 500 amplifier and pClamp9 (Axon Instruments, Union City, CA). All dissections and recordings were done in standard *Drosophila* saline at 19°C.

### Fly stocks

All animals were raised at 25°C in standard fly vials with corn meal molasses medium. Pod1 stocks were gifts from Yuh-Nung Jan (University of California, San Francisco). Control animals used were *w*^1118^.

### Data acquisition and statistics

GluR clusters were measured manually by outlining GluR clusters using NIH Image J software as previously described (Featherstone et al. 2002; Chen and Featherstone, 2005; Chen et al. 2005; Rasse et al. 2005). Total GluR fluorescence was quantified by measuring fluorescence intensity at the synapse and subtracting background/muscle fluorescence intensity using Adobe Photoshop CS2. Statistics were performed using GraphPad Prism (v. 4.01). Statistical comparisons were made using unpaired students t-tests or, for distributions, Kolmogorov-Smirnov tests. Statistical significance in figures is represented as follows: * = p < 0.05, ** = p < 0.001, and *** = p < 0.0001. All error bars represent S.E.M.

## Results

### Most PSD proteins have *Drosophila* homologs

To assess the similarity by which mammalian and fly non-NMDA receptors might be trafficked and anchored to the synapse, we searched the literature for proteins that interact with AMPARs or KARs. Of the 40 proteins we found that regulate AMPARs or KARs, 38 (95%) have *Drosophila* homologs (Table 1). If these *Drosophila* homologs function similarly to regulate GluR trafficking and localization at the glutamatergic *Drosophila* NMJ, we would expect them to be expressed in neurons, muscle, or both. Therefore, we used the Berkeley *Drosophila* Genome Project (BDGP) Gene Expression Database (http://www.fruitfly.org/cgi-bin/ex/insitu.pl) to examine the expression patterns of these genes. The expression patterns for 14 of these genes are documented. Of these, 5 are expressed in muscle, 6 are expressed in neurons, 2 are expressed ubiquitously, and one is expressed in other tissue. In other words, of the 15 genes with documented expression patterns, 93% are expressed in tissues consistent with conserved function.

Some mammalian GluRs are embedded within the PSD, a specialized protein network that allows postsynaptic cells to receive information. We extended our search of the literature to include proteins that make up the PSD. Of the 199 proteins we found that are localized to the PSD, 191 (96.0%) have *Drosophila* homologs (Supplemental Table 1). 21 of the *Drosophila* genes are homologous for more than one mammalian PSD protein, consistent with the recent confirmation that families of genes expanded between fly and mouse (Emes et al. 2008). The BDGP has documented the expression pattern for 63 of these genes. Of these, 18 are expressed in muscle, 29 are expressed in neurons, 4 are expressed in both neurons and muscle, 7 are expressed ubiquitously, and 5 are expressed in other tissues. Thus, 92% of *Drosophila* proteins homologous to mammalian PSD proteins are expressed in tissues consistent with conserved function. We conclude from these data that the signaling machinery surrounding *Drosophila* GluRs is likely to be similar to that found in the mammalian PSD.

### Mutations in *pod1* reduce GluRIIA cluster sizes

**Figure 1:**
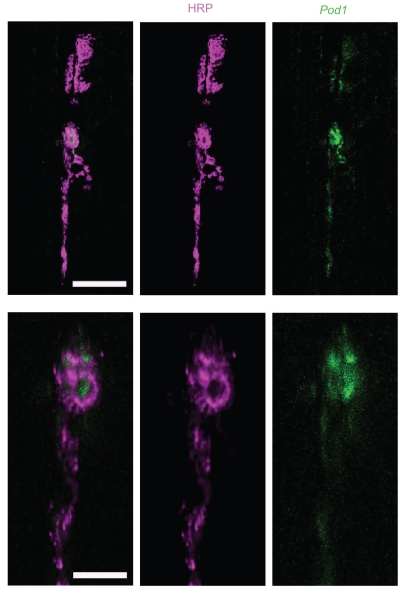
Pod1 is localized to the NMJ. Confocal fluorescent images showing NMJs on muscles 6 and 7 in wild-type third instar larvae. Animals were labeled with antibodies against HRP (magenta), which recognizes presynaptic membranes, and Pod1 (green). Scale bar in top panel = 20 μm. Bottom panels depict a high magnification view of an area from the top panels. Scale bar in bottom panels = 5 μm.

To test whether one of the *Drosophila* genes listed in Supplemental Table 1 plays a role in GluR cluster formation, we examined the NMJ of *pod1* mutants. *pod1* is one of two coronin family members in *Drosophila* and has been shown to crosslink actin and microtubules in cultured S2 cells (Rothenberg et al. 2003). We selected *pod1* for further study because the literature suggests a number of cytoskeletal proteins are part of the PSD (40 of the 199 PSD proteins in Supplemental Table 1) and *pod1* is expressed in both neurons and muscle. We first wanted to confirm that *pod1* is localized to NMJs by examining its immunoreactivity (Fig. 1) and found that Pod1 immunoreactivity (which is eliminated in *pod1* mutants; data not shown) is enriched at the NMJ suggesting Pod1 may function at the NMJ.

**Figure 2:**
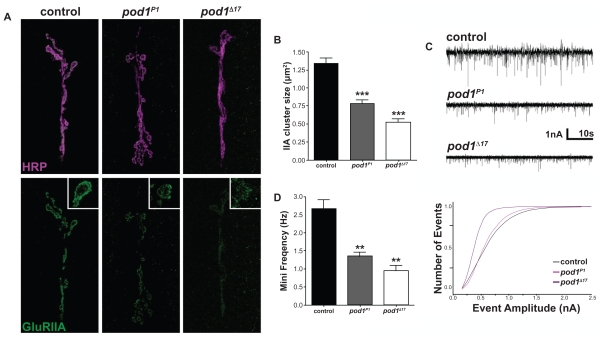
*pod1* mutants show a loss of A-type glutamate receptors. **A**) Confocal images showing the NMJ on ventral longitudinal muscles 6/7 in third instar larvae (110–120 h after egg laying), visualized using antibodies against neuronal membrane (HRP, magenta) and the glutamate receptor subunit, GluRIIA (green). **B**) Average GluRIIA cluster size was significantly reduced in *pod1* mutant third instar larvae. **C**) Representative recordings from control and *pod1* mutant third instar larve, showing spontaneous excitatory junction currents (sEJCs) in muscle 6 of the NMJ. **D**) Cumulative frequency histogram of sEJC amplitudes. *pod1* mutant animals (pink and purple traces) exhibited fewer large events, compared with control animals (black traces).

To determine whether *pod1* affects GluR cluster formation, we examined GluRs in third instar *pod1* mutants, which are viable until pupal stage (Rothenberg et al. 2003). Mutant synapses were examined immunocytochemically using α-horseradish peroxidase (HRP) to label the pre-synaptic motor neuron and α-GluRIIA to label postsynaptic GluRs (Fig. 2). α-HRP recognizes glycosylation of multiple neuronal proteins (Paschinger et al. 2008). Three mutant alleles were used for this analysis: *pod1^P{GT1}BG02604^* (hereafter referred to as *pod1^P1^*), *pod1*^Δ^*^17^*, and *pod1*^Δ^*^96^*. *pod1^P1^* contains a transposable element inserted approximately 300 bp upstream of *pod1*. The presence of the transposable element reduced Pod1 immunreactivity to undetectable levels (see above, data not shown). *pod1*^Δ^*^17^* and *pod1*^Δ^*^96^* were generated by imprecise excision of the *P{GT1}BG02604* tranposable element and remove the entire coding sequence of *pod1* (Rothenberg et al. 2003). Control animals exhibit distinct GluRIIA immunoreactivity visible as small clusters (green) opposite of the presynaptic motor neuron (magenta; Fig. 2A left panels). Each GluR punctum represents an individual postsynaptic density (Chen and Featherstone, 2005; Rasse et al. 2005; Schmid et al. 2008). GluR cluster area, measured immunocytochemically, is directly proportional to the number of GluRs measured electrophysiologically and independent of changes in NMJ morphology (Featherstone et al. 2002; Chen and Featherstone, 2005; Rasse et al. 2005; Schmid et al. 2008). All three *pod1* mutant alleles exhibited a significant reduction in GluRIIA cluster size (Fig. 2A, B and data not shown; *w^1118^* = 1.34 ± 0.07 μm^2^, n = 80 clusters from 8 animals; *pod1^P1^* = 0.79 ± 0.05 μm^2^, n = 66 clusters from 7 animals, p <0.0001; *pod1*^Δ^*^17^* = 0.53 ± 0.04 μm^2^, n = 80 clusters from 8 animals, p < 0.0001; *pod1*^Δ^*^96^* = 0.62 ± 0.06 μm^2^, n = 70 clusters from 7 animals, p <0.0001). Measurements of total fluorescence intensity indicated there is a 34% and 36% reduction in GluRIIA immunoreactivity in *pod1*^Δ^*^17^* and *pod1*^Δ^*^96^* mutant animals, respectively (normalized GluRIIA fluorescence *w^1118^* = 1.00 ± 0.16, n = 15; *pod1^P1^* = 0.69 ± 0.08, n = 9, p = 0.12; *pod1*^Δ^*^17^* = 0.66 ± 0.09, n = 14, p = 0.03; *pod1*^Δ^*^96^* = 0.64 ± 0.09, n = 9, p = 0.04). These data suggest Pod1 is involved in the expression and/or localization of GluRs.

The *Drosophila* NMJ contains two receptor types, A-type or B-type, which are differentially expressed and clustered (Marrus and DiAntonio, 2004; Schmid et al. 2008) and interact with distinct components of postsynaptic density (Chen and Featherstone, 2005; Chen et al. 2005). This raises the possibility that mutations in *pod1* may affect A-type receptors without affecting B-type receptors. To test this possibility, we examined the NMJ of *pod1*^Δ^*^17^* mutants using antibodies against either GluRIIB to label B-type receptors or GluRIIC to label all receptors. *pod1*^Δ^*^17^* mutants exhibited no difference in either GluRIIB or GluRIIC cluster sizes (GluRIIB: *w^1118^* = 0.87 ± 0.04 μm^2^, n = 90 clusters from 9 animals; *pod1*^Δ^*^17^* = 0.93 ± 0.06 μm^2^, n = 90 clusters from 9 animals, p = 0.3718; GluRIIC: *w^1118^* = 1.52 ± 0.06 μm^2^, n = 100 clusters from 10 animals; *pod1*^Δ^*^17^* = 1.47 ± 0.06 μm^2^, n = 100 clusters from 10 animals, p = 0.55). These data indicate that Pod1 affects A-type but not B-type receptors.

To determine whether the loss of A-type GluRs affects the synaptic function of the NMJ, we performed two-electrode voltage clamp. Muscle 6 was voltage clamped at −60 mV and spontaneous miniature excitatory junction currents (sEJCs or ‘minis’) were recorded. The frequency of minis is significantly reduced in *pod1* mutant animals (Fig. 2C, D; *w^1118^* = 2.7 ± 0.23 Hz, n = 10; *pod1^P1^* = 1.34 ± 0.12 Hz, n = 8, p = 0.0002; *pod1*^Δ^*^17^* = 0.95 ± 0.14 Hz, n = 7, p < 0.0001). This reduction may represent changes in presynaptic function (Rothenberg et al. 2003) as well as minis being lost in baseline noise. Consistent with this and the reduction in GluRIIA staining, sEJC amplitudes are also significantly reduced in *pod1* mutants (Fig. 2C; *pod1^P1^* K-S statistic = 0.957, p < 0.0001; *pod1*^Δ^*^17^* K-S statistic = 0.977, p < 0.0001). The smaller mini amplitudes taken together with the immunocytochemical data indicate that *pod1* mutants contain fewer A-type receptors. In agreement with this, we found that the sEJC decay time was significantly reduced in *pod1* mutants (data not shown, *w^1118^* = 12.20 ± 0.25 ms, n = 10; *pod1^P1^* = 9.96 ± 0.29 ms, n = 8, p < 0.0001; *pod1*^Δ^*^17^* = 10.76 ± 0.25 ms, n = 7, p < 0.0001). Shorter decay times are associated with specific loss of A-type GluRs (DiAntonio et al. 1999; Schmid et al. 2008). We conclude from these data that *pod1* plays a role in the expression or localization of A-type, but not B-type GluRs.

## Discussion

Synaptic plasticity and memory rely on the trafficking and proper localization of postsynaptic GluRs. Although a number of studies address the subunit-specific trafficking of AMPARs at the synapse (for reviews see Malinow and Malenka, 2002; Derkach, Oh et al. 2007; Greger et al. 2007), relatively little is known about how the receptors get transported to the synapse and anchored in the proper locations. The *Drosophila* genome encodes homologs of mammalian NMDA, AMPA, kainate, and delta receptor subunits (Sprengel et al. 2001). Therefore, an evolutionarily simpler system such as *Drosophila* could be used to dissect the function of genes and proteins that regulate GluR trafficking.

We searched the literature for proteins that regulate AMPARs or KARs and proteins that are found within the PSD. 95.8% of these proteins have *Drosophila* homologs. No homologs were found for 11 mammalian proteins. Interestingly, this included the scaffolding proteins Bassoon (Takao-Rikitsu, 2004) and AKAP 79/150 (Dell-Acqua et al. 2006). This may be due to the reduced complexity of the fly NMJ (see below).

Several lines of evidence suggest these *Drosophila* homologs may have conserved functions. First, of the homologs we examined with documented expression patterns, 92.2% are found in neurons, muscle, or both, consistent with conserved function. Further, 31 of these homologs have been reported at the *Drosophila* NMJ, which is a glutamatergic synapse. Second, 29 of the homologs were recently identified by mass spectrometry as members of a protein complex associated with the *Drosophila* NR2 GluR subunit (Emes et al. 2008). Third, two of the *Drosophila* homologs have been shown to regulate GluRs. Pak positively regulates GluR cluster formation at the NMJ when it is downstream of Dock (Albin and Davis 2004). Coracle, the *Drosophila* homolog of the mammalian 4.1 N protein (see Table 1), interacts with GluRIIA subunits and anchors A-type receptors to the actin cytoskeleton (Chen et al. 2005). Finally, four of the *Drosophila* homologs, Didum (Myosin Va), l(1)G0003 (Rab11 family interacting protein), Pnut (Cdc10 and Septin 7), and Polo (Polo-like kinase) were identified in a forward genetic screen for genes that regulate GluR cluster formation (Liebl and Featherstone, 2005) at the *Drosophila* NMJ. We present evidence here that indicates that Pod1, the *Drosophila* homolog of Coronin 7 (see Supplemental Table 1), also regulates GluR cluster formation at the *Drosophila* NMJ.

The Coronins are an evolutionarily conserved family of proteins that regulate the actin cytoskeleton and vesicle transport (for reviews see Rybakin and Clemen, 2005; Uetrecht and Bear, 2006). Mammalian Coronins 1a (Collins et al. 2006), 1b, 1c (Peng et al. 2004; Collins et al. 2006), and 2b (Jordan et al. 2004; Collins et al. 2006) were identified as components of the PSD via mass spectrometry. Coronin 7 is localized to the cis-Golgi and cytoplasmic vesicles (Rybakin et al. 2004). There are two *Drosophila* Coronin homologs. Coro is most similar to Coronins 1a, 1b, 1c, and 2b while Pod1 is most similar to Coronin 7. None of these proteins have been previously linked to GluRs. Previous studies in *Drosophila* (Rothenberg et al. 2003; Bharathi et al. 2004) and mammals (Rybakin and Clemen, 2005; Uetrecht and Bear, 2006), however, indicate that the coronins are expressed in the nervous system and/or muscle. This, coupled with their role in cytoskeleton remodeling, suggests they may be involved in GluR cluster formation. Consistent with this, we found Pod1 present at the NMJ (Fig. 1). It has also been shown to be localized in the tips of growing motor neuron axons during embryogenesis in *Drosophila* (Rothenberg et al. 2003).

We tested our hypothesis that Pod1 is involved in GluR cluster formation by examining *pod1* mutant synapses. The loss of *pod1* led to a reduction in the size of GluRIIA-containing clusters as well as a significant reduction in synaptic GluRIIA immunoreactivity. Interestingly, the GluR cluster sizes determined microscopically do not differ between *pod1^P1^* and *pod1*^Δ^*^17^* despite the fact that mini amplitudes in *pod1*^Δ^*^17^* null mutants are much lower. Surface expression of some GluRIIA may therefore be supported in *pod1^P1^* mutants even when total synaptic GluRIIA is severely reduced. A-type receptors are linked to the actin cytoskeleton via their interaction with coracle (Chen et al. 2005). This raises the possibility that the loss of GluRIIA is specific to the synapse. In this scenario, A-type receptors would be trafficked to the synapse but not properly anchored to the synapse in *pod1* mutants. Alternatively, *pod1* could be required for transport of GluRIIA-containing receptors from the cis Golgi to the synapse. Further studies will be required to determine how the loss of *pod1* affects A-type receptor trafficking.

There was no significant reduction in the sizes of GluRIIB or GluRIIC clusters. This is likely because B-type receptors are anchored to the cellular cytoskeleton in a different, unknown way. These data are consistent with the role of the coronins in mammals where they are known to regulate the actin cytoskeleton (Cai et al. 2008; for reviews see Rybakin and Clemen, 2005; Uetrecht and Bear, 2006) and suggests Coronin 7 may also participate in actin regulation. Although both A- and B-type receptors at the *Drosophila* NMJ are linked to microtubules (Liebl et al. 2005), only A-type receptors depend on the integrity of the actin cytoskeleton (Chen et al. 2005).

There exist a number of important differences between mammalian central synapses and *Drosophila* NMJ synapses. First, the *Drosophila* NMJ is a single cell *in vivo* system where a single presynaptic motor neuron synapses on a single postsynaptic muscle cell. It is estimated that mammalian CNS neurons synapse with as many as 10,000 other neurons. Therefore, the *Drosophila* NMJ is a simple model system lacking the complexity found in mammalian CNS synapses. This could partly account for the small percentage of mammalian proteins with no *Drosophila* homologs. Second, because the postsynaptic cell at the NMJ is a muscle cell, *Drosophila* NMJs lack dendritic spines but extend filopodia to contact presynaptic motor neurons during embryonic development (Ritzenthaler et al. 2000; Ritzenthaler et al. 2003). Thus, proteins and mechanisms specific to dendritic spines are probably not included at the fly NMJ. The NMJ, however, represents only a small percentage of fly glutamatergic synapses. Most fly glutamatergic synapses are found in the larval and adult CNS (Daniels et al. 2008). Consistent with this, many of the putative fly PSD proteins identified here are expressed in the fly CNS. Glutamate receptors and PSD proteins in the fly CNS probably function as in mammals. For example, similar to mammalian studies, central NMDA receptors are required for fly learning (Glanzman, 2005; Lin, 2005; Xia et al. 2005; Wu et al. 2007). It is currently unknown whether fly central synapses exhibit plasticity, but the NMJ exhibits post tetanic potentiation (Kuromi and Kidokoro, 2003; Cheung et al. 2006) and LTD (Guo and Zhong, 2006).

In conclusion, we have shown that most mammalian PSD proteins have *Drosophila* homologs and that these homologs are likely to have conserved functions. Therefore, the analysis of mutant phenotypes in *Drosophila* could enhance our understanding of GluR cluster formation and the PSD. Consistent with this, we have shown for the first time that the *Drosophila* homolog of Coronin 7, Pod1, is involved in the formation of GluRIIA containing GluR clusters possibly by regulating the actin cytoskeleton.

## Figures and Tables

**Table 1:** *Drosophila* glutamate receptor-associated protein homologs.

Protein	Accession number	Proposed function	*Drosophila* homolog	% Identity/positives
4.1 N	Q9H4G0	May provide a link between AMPARs and the cytoskeleton by binding to GluR1 (Shen et al. 2000)	Cora	57.8/73.5
AMPAR Binding Protein	AF090113.1	Protein scaffold that binds to the PDZ domain of GluR2 (Srivastava and Ziff, 1999)	GRIP	30.9/49.0
AKAP 79/150	NM_133515.1	Anchor kinases and phosphatases and binds to SAP97 (Colledge et al. 2000)	None	
Adenomatous polyposis coli (APC)	NM_000038.3	Involved in AMPAR clustering possibly by its interaction with PSD-95 (Senda et al. 2005; Shimomura et al. 2007)	APC	53.1/63.5
AP-2, μ2	NM_001025205.1	Binds to cytoplasmic tail of AMPARs to promote endocytosis of receptors (Osterweil et al. 2005; Kastning et al. 2007)	AP-50	87.2/94.3
Actinfilin	NM_145671.1	Targets KARs for degradationn by binding to both GluR6 and Cullin 3 (Salinas et al. 2006)	CG15097	54.4/71.4
β-catenin	NM_007614.2	Forms a complex with N-cadherin and AMPARs possibly regulating surface expression of AMPARs (Nuriya and Huganir, 2006)	Arm	66.3/76.7
cGMP-dependent protein kinase II (cGKII)	Z36276.1	Increases extrasynaptic surface expression of AMPARs by binding to GluR1 CTD (Serulle et al. 2007)	For Pkg21D	50.2/68.7 45.4/64.1
Dynamin-3	NM_015569.2	Maintains level of synaptic AMPARs by positioning endocytic proteins near the PSD (Lu et al. 2007)	Shi	69.6/81.9
GIT-1	Q9Z272	Involved in AMPAR trafficking by forming a complex with AMPARs, KIF1A, GRIP, and liprin-α (Shin et al. 2003; Lu et al. 2007)	CG16728	44.1/59.2
GRIP-associated protein 1 (GRASP-1)	NM_207672.1	RasGEF that binds to GRIP and JNK and regulates synaptic targeting of AMPARs (Ye et al. 2000; Ye et al. 2007)	CG31784	25.2/47.3
GRIP	NM_021150.1	Scaffolding protein that binds to GluR2 and GluR3 (Dong et al. 1997)	GRIP	56.1/81.7
Hsp90	S45392.1	Required for constitutive cycling of AMPARs (Gerges et al. 2004b)	Hsp83	70.7/79.7
JNK	AB005665.1	Acts on GluR2 (long isoform) and GluR4 to regulate cell surface expression of AMPARs (Zhu et al. 2005; Thomas et al. 2008)	Bsk	77.7/87.2
KIF1A	Q12756	Involved in AMPAR trafficking by forming a complex with AMPARs, GIT-1, GRIP, and liprin-α (Shin et al. 2003; Lu et al. 2007)	Unc-104	55.2/68.7
KIF17	AB001424.1	Required for localization of KARs by binding to GluR6 and KA2 (Kayadjanian et al. 2007)	Klp64D	57.5/71.2
Kalirin	NM_032062.1	RhoGEF that interacts with GluR1 and regulates AMPAR insertion in response to activity (Xie et al. 2007)	Trio	41.6/60.7
KRIP6	Q56A24	Regulates KARs by binding to GluR6 (Laezza et al. 2007)	Dbo CG3571	35.9/50.6 32.6/50.2
Lin-10	NM_025187.3	Involved in AMPAR trafficking by binding to PDZ domain (Stricker and Huganir, 2003)	CG7083	51.9/67.9
Liprin-α	BC034046.1	Involved in AMPAR trafficking by forming a complex with AMPARs, KIF1A, GIT-1, and GRIP (Shin et al. 2003; Lu et al. 2007)	Liprin-α	47.8/60.2
Myosin Va	NM_000259.2	Required for transport of AMPARs during synaptic activity (Correia et al. 2008)	Didum	39.5/57.6
Myosin Vb	NM_001080467.1	Regulates AMPAR surface expression by associating with GluR1 (Lise et al. 2006)	Didum	42.8/60.3
Myosin VI	NM_004999.3	Involved in AMPAR endocytosis (Osterweil et al. 2005) and forms a complex with GluR1 and SAP-97 (Wu et al. 2002)	Jar	53.2/71.5
N-cadherin	AB017695.1	Forms a complex with neural plakophilin-related arm protein (NPRAP), ABP, and GRIP to anchor AMPARs (Silverman et al. 2007)	CadN	29.0/44.4
Neuronal-activity related pentraxin (NARP)	S82649.1	Associate with GluR1- containing AMPARs and may play a role in clustering of AMPARs (O’Brien et al. 1999; O’Brien et al. 2002)	B6	29.9/46.2
NEEP21	NM_024128.3	Component of neuronal endosomes that is necessary for the recycling of AMPARs (Steiner et al. 2005; Kulangara et al. 2007)	None	
NPRAP	Q9UQB3	Forms a complex with N-cadherin, ABP, and GRIP to anchor AMPARs (Silverman et al. 2007)	P120ctn	46.2/62.9
NSF	AL603829.5	Promotes constitutive cycling of AMPARs (Nishimune et al. 1998) by disrupting GluR2 and PICK1 (Hanley et al. 2002)	Nsf2 Comt	60.1/74.4 59.7/74.0
PICK1	AB026491.1	Promotes internalization of GluR2-containing AMPARs (Perez et al. 2001; Terashima et al. 2004)	PICK1	60.8/76.3
Rab8	AF498943.1	Involved in constitutive cycling and delivery of AMPARs to membrane surface (Gerges et al. 2004a; Brown et al. 2007)	Rab8	79.2/88.4
Rab11	P62494	Responsible for delivery of GluR1-containing receptors to the synapse (Park et al. 2004; Brown et al. 2007)	Rab11	85.5/90.2
RIL	Y08361.1	Links internalized GluR1- containing receptors to actin cytoskeleton (Schulz et al. 2004)	CG30084	41.2/51.0
SAP97	NM_012788.1	Scaffolding protein that binds to GluR1 (Leonard et al. 1998)	Dlg1	53.9/68.6
Shank	AF133301.1	Scaffolding protein that helps position AMPAR endocytic machinery at the PSD (Lu et al. 2007)	Prosap	50.6/67.2
SNAP (β isoform)	P28663	Mediates disassembly of GluR2-PICK1 complex (Hanley et al. 2002)	Snap	61.6/78.2
SUMO	P63166	Modifies GluR6 to promote endocytosis of KARs (Martin et al. 2007)	Smt3	52.3/70.5
SynGAP	NM_001113409.1	Involved in AMPAR trafficking to synapse (Rumbaugh et al. 2006)	CG32560	37.7/55.1
γ2 (Stargazin)	NM_006078.2	Involved in localization of AMPARs to synapse and delivery to cell surface (Chen et al. 2000)	Stg1	26.1/38.9
γ3 (TARP)	NM_006539.2	Required for expression of AMPARs on cell surface (Tomita et al. 2003)	Stg1	27.7/42.9
γ8 (TARP)	NM_ 080696.2	Required for expression of AMPARs on cell surface (Tomita et al. 2003)	Stg1	28.9/43.0

References Table 1

Brown, T.C., Correia, S.S., Petrok, C.N. et al. 2007. Functional compartmentalization of endosomal trafficking for the synaptic delivery of AMPA receptors during long-term potentiation. *J. Neurosci.*, 27:13311–15.

Chen, L., Chetkovich, D.M., Petralia, R.S. et al. 2000. Stargazin regulates synaptic targeting of AMPA receptors by two distinct mechanisms. *Nature*, 408:936–43.

Colledge, M., Dean, R.A., Scott, G.K. et al. 2000. Targeting of PKA to glutamate receptors through a MAGUK-AKAP complex. *Neuron*, 27:107–19.

Correia, S.S., Bassani, S., Brown, T.C. et al. 2008. Motor protein-dependent transport of AMPA receptors into spines during long-term potentiation. *Nat. Neurosci.*, 11:457–66.

Dong, H., O’Brien, R.J., Fung, E.T. et al. 1997. GRIP: a synaptic PDZ domain-containing protein that interacts with AMPA receptors. *Nature*, 386:279–84.

Gerges, N.Z., Backos, D.S. and Esteban, J.A. 2004a. Local control of AMPA receptor trafficking at the postsynaptic terminal by a small GTPase of the Rab family. *J. Biol. Chem.*, 279:43870–78.

Gerges, N.Z., Tran, I.C., Backos, D.S. et al. 2004b. Independent functions of hsp90 in neurotransmitter release and in the continuous synaptic cycling of AMPA receptors. *J. Neurosci.*, 24:4758–66.

Hanley, J.G., Khatri, L., Hanson, P.I. et al. 2002. NSF ATPase and alpha-/beta-SNAPs disassemble the AMPA receptor-PICK1 complex. *Neuron*, 34:53–67.

Kastning, K., Kukhtina, V., Kittler, J.T. et al. 2007. Molecular determinants for the interaction between AMPA receptors and the clathrin adaptor complex AP-2. *Proc. Natl. Acad. Sci. U.S.A.*, 104:2991–6.

Kayadjanian, N., Lee, H.S., Pina-Crespo, J. et al. 2007. Localization of glutamate receptors to distal dendrites depends on subunit composition and the kinesin motor protein KIF17. *Mol. Cell Neurosci.*, 34:219–30.

Kulangara, K., Kropf, M., Glauser, L. et al. 2007. Phosphorylation of glutamate receptor interacting protein 1 regulates surface expression of glutamate receptors. *J. Biol. Chem.*, 282:2395–404.

Laezza, F., Wilding, T.J., Sequeira, S. et al. 2007. KRIP6: a novel BTB./kelch protein regulating function of kainate receptors. *Mol. Cell Neurosci.*, 34:539–50.

Leonard, A.S., Davare, M.A., Horne, M.C. et al. 1998. SAP97 is associated with the alpha-amino-3-hydroxy-5-methylisoxazole-4-propionic acid receptor GluR1 subunit. *J. Biol. Chem.*, 273:19518–24.

Lise, M.F., Wong, T.P., Trinh, A. et al. 2006. Involvement of myosin Vb in glutamate receptor trafficking. *J. Biol. Chem.*, 281:3669–78.

Lu, J., Helton, T.D., Blanpied, T.A. et al. 2007. Postsynaptic positioning of endocytic zones and AMPA receptor cycling by physical coupling of dynamin-3 to Homer. *Neuron*, 55:874–89.

Martin, S., Nishimune, A., Mellor, J.R. et al. 2007. SUMOylation regulates kainate-receptor-mediated synaptic transmission. *Nature*, 447:321–5.

Nishimune, A., Isaac, J.T., Molnar, E. et al. 1998. NSF binding to GluR2 regulates synaptic transmission. *Neuron*, 21:87–97.

Nuriya, M. and Huganir, R.L. 2006. Regulation of AMPA receptor trafficking by N-cadherin. *J. Neurochem.*, 97:652–61.

O’Brien, R., Xu, D., Mi, R. et al. 2002. Synaptically targeted narp plays an essential role in the aggregation of AMPA receptors at excitatory synapses in cultured spinal neurons. *J. Neurosci.*, 22:4487–98.

O’Brien, R.J., Xu, D., Petralia, R.S. et al. 1999. Synaptic clustering of AMPA receptors by the extracellular immediate-early gene product Narp. *Neuron*, 23:309–23.

Osterweil, E., Wells, D.G. and Mooseker, M.S. 2005. A role for myosin VI in postsynaptic structure and glutamate receptor endocytosis. *J. Cell Biol.*, 168:329–38.

Park, M., Penick, E.C., Edwards, J.G. et al. 2004. Recycling endosomes supply AMPA receptors for LTP. *Science*, 305:1972–5.

Rumbaugh, G., Adams, J.P., Kim, J.H. et al. 2006. SynGAP regulates synaptic strength and mitogen-activated protein kinases in cultured neurons. *Proc. Natl. Acad. Sci. U.S.A.*, 103:4344–51.

Salinas, G.D., Blair, L.A., Needleman, L.A. et al. 2006. Actinfilin is a Cul3 substrate adaptor, linking GluR.6 kainate receptor subunits to the ubiquitin-proteasome pathway. *J. Biol. Chem.*, 281:40164–73.

Schulz, T.W., Nakagawa, T., Licznerski, P. et al. 2004. Actin/alpha-actinin-dependent transport of AMPA receptors in dendritic spines: role of the PDZ-LIM protein RIL. *J. Neurosci.*, 24:8584–94.

Senda, T., Shimomura, A. and Iizuka-Kogo, A. 2005. Adenomatous polyposis coli (Apc) tumor suppressor gene as a multifunctional gene. *Anat. Sci. Int.*, 80:121–31.

Serulle, Y., Zhang, S., Ninan, I. et al. 2007. A GluR1-cGKII interaction regulates AMPA receptor trafficking. *Neuron*, 56:670–88.

Shen, L., Liang, F., Walensky, L.D. et al. 2000. Regulation of AMPA receptor GluR.1 subunit surface expression by a 4. 1N-linked actin cytoskeletal association. *J. Neurosci*, 20:7932–40.

Shimomura, A., Ohkuma, M., Iizuka-Kogo, A. et al. 2007. Requirement of the tumour suppressor APC for the clustering of PSD-95 and AMPA receptors in hippocampal neurons. *Eur. J. Neurosci.*, 26:903–12.

Shin, H., Wyszynski, M., Huh, K.H. et al. 2003. Association of the kinesin motor KIF1A with the multimodular protein liprin-alpha. *J. Biol. Chem.*, 278:11393–401.

Silverman, J.B., Restituito, S., Lu, W. et al. 2007. Synaptic anchorage of AMPA receptors by cadherins through neural plakophilin-related arm protein AMPA receptor-binding protein complexes. *J. Neurosci.*, 27:8505–16.

Srivastava, S. and Ziff, E.B. 1999. ABP: a novel AMPA receptor binding protein. *Ann. N.Y. Acad. Sci.*, 868:561–4.

Steiner, P., Alberi, S., Kulangara, K. et al. 2005. Interactions between NEEP21, GRIP1 and GluR2 regulate sorting and recycling of the glutamate receptor subunit GluR2. *Embo J.*, 24:2873–84.

Stricker, N.L. and Huganir, R.L. 2003. The PDZ domains of mLin-10 regulate its trans-Golgi network targeting and the surface expression of AMPA receptors. *Neuropharmacology*, 45:837–48.

Terashima, A., Cotton, L., Dev., K.K. et al. 2004. Regulation of synaptic strength and AMPA receptor subunit composition by PICK1. *J. Neurosci.*, 24:5381–90.

Thomas, G.M., Lin, D.T., Nuriya, M. et al. 2008. Rapid and bi-directional regulation of AMPA receptor phosphorylation and trafficking by JNK. *Embo J.*, 27:361–72.

Tomita, S., Chen, L., Kawasaki, Y. et al. 2003. Functional studies and distribution define a family of transmembrane AMPA receptor regulatory proteins. *J. Cell Biol.*, 161:805–16.

Wu, H., Nash, J.E., Zamorano, P. and Garner, C.C. 2002. Interaction of SAP97 with minus-end-directed actin motor myosin VI. Implications for AMPA receptor trafficking. *J. Biol. Chem.*, 277:30928–34.

Xie, Z., Srivastava, D.P., Photowala, H. et al. 2007. Kalirin-7 controls activity-dependent structural and functional plasticity of dendritic spines. *Neuron*, 56:640–56.

Ye, B., Yu, W.P., Thomas, G.M. et al. 2007. GRASP-1 is a neuronal scaffold protein for the JNK signaling pathway. *FEBS Lett.*, 581:4403–10.

Ye, B., Liao, D., Zhang, X. et al. 2000. GRASP-1: a neuronal RasGEF associated with the AMPA receptor/GRIP complex. *Neuron*, 26:603–17.

Zhu, Y., Pak, D., Qin, Y. et al. 2005. Rap2-JNK removes synaptic AMPA receptors during depotentiation. *Neuron*, 46:905–16.

## Abbreviations

BDGP: Berkeley Drosophila Genome Project
GluRs: glutamate receptors
HRP: horseradish peroxidase
LTD: long term depression
LTP: long term potentiation
NMDA: N-methyl-D-aspartate
NMJ: neuromuscular junction
PSD: postsynaptic density
TARPs: transmembrane AMPA receptor regulatory proteins
KARs: kainate receptors
